# Panoramic radiography and cone-beam computed tomography findings in preoperative examination of impacted mandibular third molars

**DOI:** 10.1186/1472-6831-14-71

**Published:** 2014-06-14

**Authors:** Ilkay Peker, Cigdem Sarikir, Meryem Toraman Alkurt, Zeynep Fatma Zor

**Affiliations:** 1 Department of Dentomaxillofacial Radiology, Gazi University Faculty of Dentistry, 82. Sok No: 4 06510, Emek-Ankara, Turkey; 2 Department of Oral and Maxillofacial Surgery, Gazi University Faculty of Dentistry, Ankara, Turkey

**Keywords:** Cone-beam computed tomography, Digital panoramic radiography, Impacted mandibular third molars, Inferior alveolar canal

## Abstract

**Background:**

Preoperative radiographic examination of impacted mandibular third molars (IMTM) is essential to prevent inferior alveolar nerve injury during extraction. The purpose of this study was to evaluate the correlation between cone-beam computed tomography (CBCT) and digital panoramic radiography (DPR) findings in preoperative examination of IMTM.

**Methods:**

This retrospective study included 298 teeth in 191 individuals. The relationship between the inferior alveolar canal (IAC) and the IMTM (buccal, lingual, interradicular or inferior), the position of the IMTM with respect to the IAC (contact, no contact), the morphologic shape of the mandible in the IMTM region (round, lingual extended, lingual concave), the type of IMTM (vertical, horizontal or angular) and the number of roots of the IMTM were evaluated on CBCT images. DPR images were evaluated for the number of roots of the IMTM and for the most common radiographic findings indicating a relationship between the IAC and the IMTM (darkening of the roots, diversion of the IAC, narrowing of the IAC and interruption of the white line). Data were statistically analyzed with Cramer V coefficient, Kappa statistic, chi-square and Fisher’s exact test.

**Results:**

There was a significant difference in number of roots detected on DPR versus CBCT images. There was a significant association between the type of IMTM and the morphologic shape of the mandible on CBCT images. Darkening of the roots and interruption of the white line on DPR images were significantly associated with the presence of contact between the IMTM and the IAC on CBCT images.

**Conclusions:**

Panoramic radiography is inadequate, whereas CBCT is useful to detect multiple roots of IMTM. When darkening of the roots and interruption of the white line are observed on panoramic images, there is increased likelihood of contact between the IMTM and the IAC. CBCT is required in these cases.

## Background

Extraction of impacted mandibular third molars (IMTM) is a routine procedure in oral surgery, with several possible postoperative complications [1]. The most common complications are injury to the inferior alveolar nerve (IAN) or to the lingual nerve, dysesthesia and lingual fracture of the mandible [2]-4]. The incidence of temporary IAN injury related to extraction of IMTM varies from 0.4% to 9.4% [5-8]. In contrast, the rate of permanent IAN injury is reported to be less than 1% [9]. Factors that increase the risk of nerve damage include close proximity between the third molars and the inferior alveolar canal (IAC) and the presence of direct contact between the tooth roots and the IAN [10-12]. Some authors have reported that the most important factor for IAN injury is the anatomical relationship between the impacted third molar and the IAC [13,14]. Additionally, it has been reported that factors such as surgeons’ experience, operative procedures, institutional settings, and anatomical and radiographic factors can affect the likelihood of IAC damage [8,15].

Accurate assessment of the relationship between the IMTM and the IAC before surgery is necessary to avoid IAN injury [16]. Panoramic radiography is frequently used as the standard diagnostic imaging method for this purpose in clinical practice [17]. In many cases, panoramic images are sufficient for preoperative assessment of IMTM; however, this technique cannot provide any information about the buccolingual direction [18]. Assessment of the buccolingual direction is very important for cases in which the IMTM and the IAC are in close proximity [18,19]. Three-dimensional (3D) imaging with conventional computed tomography and cone-beam computed tomography (CBCT) is recommended in these cases to detect the exact relationship [18].

CBCT was developed for dentomaxillofacial imaging because it produces a lower radiation dose with high spatial resolution, is affordable and requires less space than conventional computed tomography [17-20]. Previous studies have reported that CBCT is more accurate than conventional methods such as panoramic radiography for determining the relationship between impacted third molars and the IAC [17,21-24].

This study provides information to assist clinicians in deciding when CBCT is required in the preoperative examination of IMTM. The study’s purpose was to describe and evaluate the correlation between CBCT and digital panoramic radiography (DPR) findings in detecting the number of roots of IMTM and the relationship between panoramic signs and the presence of contact between the IMTM and the IAC.

## Methods

### Patients

This retrospective study was approved by the Ethical Board of the Institutional Ethics Committee of Ankara University Faculty of Dentistry (Ankara, Turkey). Informed consent was obtained from all volunteers. This study included 298 teeth in 191 individuals applied to our clinic between January 2011 and October 2013. The patients underwent preoperative radiographic examination to evaluate the relationship between IMTM and the IAC. IMTM associated with any pathology such as cysts or tumors were excluded from the study.

### Imaging

DPR images were obtained using a Veraviewpocs 2D unit (J. Morita Mfg. Corp., Kyoto, Japan), operating at 60–90 kVp, 1–10 mA, with a 0.5 mm focal spot and an exposure time of 7.4 seconds. CBCT images were obtained using a Promax 3D unit (Planmeca Oy, Helsinki, Finland), operating at 84 kVp, 9–14 mA, with a 0.16 mm voxel size, an exposure time of 6 seconds and a field of view of 8 cm.

### Image evaluation

DPR and CBCT images were evaluated independently by two oral radiologists with at least 2 years of experience on the computer monitor (21 inch LCD monitor with 1280 × 1024 resolution) in a quiet room with subdued ambient lighting. The observers were allowed to manipulate the contrast and brightness features and to use the software’s zoom tool. Because this was a retrospective study and the patients had been scanned with standard settings, the dataset could not be reoriented as suggested by Lübbers et al., who reported that oblique plane scanning, which needs a small number of slices with a relatively small volume, can be helpful if the IAC is difficult to visualize [19,25]. After 30 days, all images were re-evaluated by the same two observers.

The number of tooth roots (1, 2, 3 or 4) was evaluated on DPR and CBCT images. Roots were considered to be separate when the furcation was located in the cervical or middle third of the roots [22]. The relationship between the IMTM and the IAC was evaluated on panoramic images according to criteria established by Rood and Shehab [26]. In the present study, the most common radiographic findings (darkening of the roots, diversion of the mandibular canal, narrowing of the mandibular canal and interruption of the white line) were examined as previously described by Szalma et al. [27].

IMTM were classified into three types based on their orientation on cross-sectional slices of CBCT images: type A, vertical (impacted teeth oriented in an upright position, 90° to the mandible); type B, horizontal (impacted teeth oriented parallel to the mandible); and type C, angular (teeth angled in a forward/backward position or < 90° to the mandible) [21]. The buccolingual relationship between the IMTM and the IAC was classified as buccal, lingual, interradicular or inferior [23]. The position of the IAC with respect to the third molar was classified as contact (no bone between the IAC and the third molar) or no contact (bone between the IAC and the third molar). The morphologic shape of the bone in the third molar region was classified as: type 1, round (round on both buccal and lingual sides); type 2, lingual extended (slightly straight on the buccal side with a bony extension on the lingual side); and type 3, lingual concave (lingual concave on the lingual side and round on the buccal side) [21].

### Data analysis

Obtained data were statistically analyzed with descriptive statistics, Cohen’s Kappa statistic, chi-square and Fisher’s exact tests. The Cramer V coefficient was calculated for intraobserver agreement and Cohen’s Kappa statistic was used to assess interobserver agreement. These methods were interpreted as follows: less than or equal to 0.40, poor agreement; 0.40–0.59, moderate agreement; 0.60–0.74, good agreement; 0.75–1.00, excellent agreement. Correlation between DPR and CBCT images was evaluated by chi-square and Fisher’s exact tests, with a significance level of p < 0.05.

## Results

The study comprised 123 women (64.4%) and 68 men (35.6%) between 19 and 61 years of age (mean 30.1 years). Intraobserver agreement was excellent for all variables and for both imaging methods, according to Cramer V coefficient (Observer 1: 0.904 for DPR and 0.964 for CBCT; Observer 2: 0.946 for DPR and 0.962 for CBCT). Interobserver agreement was moderate for the number of roots on DPR images (0.632) and was excellent (0.888) for the other variables, according to Cohen’s Kappa coefficient.

There was a significant difference in the number of third molar tooth roots detected on DPR versus CBCT images (p < 0.05; Table 1). There was a significant association between darkening of the roots and interruption of the white line on DPR images and the presence of contact between the IMTM and the IAC on CBCT images (p < 0.05). No significant association was found between other variables on DPR images and the position of the IAC-IMTM on CBCT images (p > 0.05; Table 2). There was a significant association (p < 0.05) between type of IMTM and the morphologic shape of the mandible on CBCT images (Table 3). Angular IMTM were most often found in patients with round and lingual extended mandibles, while vertical IMTM were most often found in lingual concave mandibles. The IAC was most often located on the lingual side of the IMTM, and there was often contact between the IMTM and the IAC. However, no statistically significant difference was found (p > 0.05) between the position of the IAC-IMTM and the buccolingual relationship of the IAC-IMTM on CBCT images (Table 4).

**Table 1:** Relationship between DPR and CBCT findings for the number of roots

**The number of roots on DPR images**		**The number of roots on CBCT images**			**Total**	**Chi-square**	**P value**
		**1 root**	**2 roots**	**3 roots**	**Total**		
**1 root**	n	29	14	1	44	159.579	0.000^*^
	%	65.9%	31.8%	2.3%	100.0%		
**2 roots**	n	2	223	28	253		
	%	0.8%	88.1%	11.1%	100.0%		
**3 roots**	n	0	0	1	1		
	%	0.0%	0.0%	100.0%	100.0%		
**Total**	n	31	237	30	298		
	%	10.4%	79.5%	10.1%	100.0%		

DPR: Digital panoramic radiography.

CBCT: Cone-beam computed tomography.

^*^Statistically significant difference p < 0.05.

**Table 2:** Relationship of the variables between DPR and CBCT images

			**Variables for DPR images**					**Total**	**Chi-square**	**P value**
**Variables for CBCT images**			**Interruption in white line of IAC**	**Diversion of IAC**	**Narrowing of IAC**	**Darkening of roots**	**Other**			
**Types of IMTM**	**Type A**	n	41	8	8	40	5	102	10.4783	0.233
		%	40.2	7.8	7.8	39.2	4.9	100.0		
	**Type B**	n	32	5	11	20	2	70		
		%	45.71	7.14	15.71	28.57	2.86	100.00		
	**Type C**	n	38	12	13	59	4	126		
		%	30.16	9.52	10.32	46.83	3.17	100.00		
	**Total**	n	111	25	32	119	11	298		
		%	37.25	8.39	10.74	39.93	3.69	100.00		
**Buccolingual relationship of IAC-IMTM**	**Buccal**	n	21	2	4	21	1	49	11.351	0.499
		%	42.9	4.1	8.2	42.9	2.0	100.0%		
	**Lingual**	n	68	16	22	80	9	195		
		%	34.9	8.2	11.3	41.0	4.6	100.0%		
	**Interradicular**	n	5	0	1	7	0	13		
		%	38.5	0.0	7.7	53.8	0.0	100.0%		
	**Inferior**	n	17	7	5	11	1	41		
		%	41.5	17.1	12.2	26.8	2.4	100.0%		
	**Total**	n	111	25	32	119	11	298		
		%	37.2	8.4	10.7	39.9	3.7	100.0%		
**Position of IAC-IMTM**	**Contact**	n	61	23	27	101	1	213	54.113	0.000^*^
		%	28.6	10.8	12.7	47.4	0.5	100.0%		
	**No contact**	n	50	2	5	18	10	85		
		%	58.8	2.4	5.9	21.2	11.8	100.0%		
	**Total**	n	111	25	32	119	11	298		
		%	37.2	8.4	10.7	39.9	3.7	100.0%		
**Mandible shape**	**Type 1**	n	46	13	15	62	1	137	13.720	0.089
		%	33.6	9.5	10.9	45.3	0.7	100.0%		
	**Type 2**	n	13	1	4	5	1	24		
		%	54.2	4.2	16.7	20.8	4.2	100.0%		
	**Type 3**	n	52	11	13	52	9	137		
		%	38.0	8.0	9.5	38.0	6.6	100.0%		
	**Total**	n	111	25	32	119	11	298		
		%	37.2	8.4	10.7	39.9	3.7	100.0%		

DPR: Digital panoramic radiography.

CBCT: Cone-beam computed tomography.

IAC: Inferior alveolar canal.

IMTM: Impacted mandibular third molar.

^*^Statistically significant difference p < 0.05.

**Table 3:** Relationship between type of IMTM and morphologic shape of the mandible on CBCT images

**Shape of the mandible**		**Types of IMTM**				**Chi-square**	**P value**
		**Vertical**	**Horizontal**	**Angular**	**Total**		
**Round**	n	35	34	68	137	11.061	0.026^*^
	%	25.5%	24.8%	49.6%	100.0%		
**Lingual extended**	n	7	7	10	24		
	%	29.2%	29.2%	41.7%	100.0%		
**Lingual concave**	n	60	29	48	137		
	%	43.8%	21.2%	35.0%	100.0%		
**Total**	n	102	70	126	298		
	%	34.2%	23.5%	42.3%	100.0%		

^*^Statistically significant difference p < 0.05.

IMTM: Impacted mandibular third molar.

**Table 4:** Relationship between the position of the IAC-IMTM and the buccolingual position of the IAC-IMTM on CBCT images

**Position of IAC-IMTM**		**Buccolingual relationship IAC-IMTM**					**Chi-square**	**P value**
		**Buccal**	**Lingual**	**Interradicular**	**Inferior**	**Total**		
**Contact**	n	32	144	12	25	213	6.437	0.092
	%	15.0%	67.6%	5.6%	11.7%	100.0%		
**No contact**	n	17	51	1	16	85		
	%	20.0%	60.0%	1.2%	18.8%	100.0%		
**Total**	n	49	195	13	41	298		
	%	16.4%	65.4%	4.4%	13.8%	100.0%		

IAC: Inferior alveolar canal.

IMTM: Impacted mandibular third molar.

## Discussion

IAN injury is a serious complication during extraction of mandibular third molars. Risk factors for injury include surgeon’s experience, age and sex of the patient, operative tissue damage, postoperative edema and surgical procedures [10]. It has been reported that the most important factor for IAN injury is the anatomical relationship between the impacted third molar and the IAC [13,14]. However, other authors have emphasized that multiple factors, including surgeon’s experience, surgical technique, institutional setting, and anatomical and radiographic factors are associated with an increased risk of IAC damage [8,15].

Accurate preoperative evaluation is necessary for successful surgery because the oral surgeon must know the angle and/or type of impacted third molar to select a suitable procedure and to prevent IAN injury and perforation and fracture of the mandible [10]. Panoramic radiography is a standard diagnostic tool for initial assessment of the relationship between the IMTM and the IAC. Because this method produces two-dimensional images, it cannot provide information in axial, coronal and sagittal planes [10]. CBCT is a more reliable imaging method in the preoperative assessment of mandibular third molars [10,15,17,19,21,24,27]. In this study, the correlations between preoperative DPR and CBCT findings and intra- and interobserver agreement were investigated.

The number of mandibular third molar roots visible on DPR versus CBCT images has been investigated in relatively few studies [22,28]. These studies reported that panoramic radiography has limited accuracy in determining the number of roots and that CBCT is more reliable for this purpose [22,28]. In the present study, intraobserver agreement was excellent for all variables on both DPR and CBCT images, whereas interobserver agreement was moderate for detection of number of roots on DPR images. Also, there was a statistically significant difference in number of roots detected on DPR versus CBCT images. The results demonstrated that DPR images were inadequate to detect multiple roots of IMTM. This result is in agreement with previous studies [22,28].

Several studies have reported that the risk of IAN injury increases when specific findings are observed on panoramic images taken to determine the relationship between third molars and the IAC [4,24,29-33]. These findings include darkening of the roots and interruption of the white line of the IAC [24,29-35]. Eyrich et al. reported that narrowing of the IAC increased the risk of IAN impairment [8]. The probability of contact between third molar roots and the IAC was higher in cases with the abovementioned signs on panoramic images [24,29-36]. Gomes et al. reported no statistically significant association between the presence of panoramic radiographic signs and IAN paresthesia after third molar extraction [29]. However, Ghaeminia et al. found that there was a significant association between panoramic radiographic signs and IAN exposure [36], a finding that has been supported by several authors [24,27,35,36]. These authors agreed that CBCT is useful for the assessment of IMTM in the buccolingual direction. In the present study, contact between the third molar roots and the IAC was most often detected on CBCT images in cases with interruption of the white line of the IAC and darkening of the roots on DPR images, a finding in agreement with several previous studies [12,24,27,29-32,34].

Oral surgeons must know the type and/or angle of the impacted third molar before surgery to prevent perforation and fracture of the mandible, and to select appropriate operation procedures [34]. Previous studies have classified the IMTM as vertical, horizontal or angular, based on its orientation to the mandible [21,32]. Tantanapornkul et al. reported that the horizontal type was the most frequent (52%), followed by angular (32%) and vertical (16%) [32]. Momin et al. reported similar results, with 42% horizontal, 37% angular and 21% vertical [21]. Msagati et al. and Syed et al. found that the mesioangular type was the most common (76% in Msagati’s study and 50.75% in Syed’s study) [37,38]. Lübbers et al. reported that mesially angulated (40.2%) and vertical (29%) were the most common types [19]. In the present study, the most frequent type was found to be angular (42.28%), followed by vertical (34.24%) and horizontal (23.48%). This finding was in agreement with the results of Lübbers et al. [19]. Differences between studies may arise from different study samples.

The shape of the mandible is an important factor in determining the use of elevators during surgery to avoid direct or indirect pressure on the IAN and perforation or fracture of the bone [21,39]. Two-dimensional images cannot provide information about bone morphology. Preoperative palpation of the related region and 3D imaging are necessary to determine the shape of the mandible [39-41] and to provide important information for the oral surgeon during elevation. The shape of the posterior mandible has been described in the literature by different classifications for various purposes. The posterior mandible has been categorized as convex, parallel or undercut for implant placement [39-41]. Watanabe et al. classified the mandible as round, lingual concave or buccal concave, and reported that the round shape (61%) was the most common [39]. In contrast, Lin et al. found that the least common type was round (21%) in the posterior mandible [41]. Momin et al. classified the mandible as round, lingual extended or lingual concave to assess bone morphology in the third molar region for preoperative planning. They reported that the round shape (49%) was the most common, followed by lingual concave (32%) and lingual extended (18%) [21]. The authors also investigated the correlation between impaction type and mandibular shape and reported that there was no significant association between these variables. In this study, mandibular shape was classified into three types: round, lingual extended and lingual concave. The prevalence of round and lingual concave types was equal (approximately 46%) and the lingual extended was least common (approximately 8%). This finding is in agreement with the results of Momin et al. [21]. Different mandibular shape prevalence in different studies can arise from racial features. There was a significant difference in impaction type according to mandible shape in this study, in contrast to the findings of Momin et al. [21].

Surgeon’s knowledge about the location of the IAN is very important in the preoperative evaluation of impacted third molars to direct the elevator and luxate the involved tooth. IAN injuries commonly occur during third molar removal because of compression and traction on the nerve through movements of the tooth roots [6]. IAN injury may occur during elevation of mesioangular impacted third molars because the roots may move downward and may compress the nerve [23]. Also, movements of the third molar root in the buccolingual direction can cause compression of the IAN. The surgical approach is generally started on the buccal side of the impacted third molar in cases in which the surgeon lacks information about the buccolingual course of the IAN before surgery. However, the IAN may experience undesirable forces if it is positioned lingually, and IAN injury has been reported in such cases [17,23]. CBCT images allow the clinician to perform comprehensive treatment planning and surgical method selection during preoperative assessment [23]. Previous studies have reported that the IAC is most frequently positioned on the lingual side of impacted third molars and that contact between the IAC and the impacted teeth was generally observed in those cases [10,17,23,34]. In the present study, the IAC was most frequently located on the lingual side of the IMTM and they were commonly in contact.

## Conclusions

This study revealed that the number of roots cannot be determined accurately on panoramic radiography images. CBCT is useful to detect multiple roots of IMTM. When darkening of the roots and interruption of the white line were observed on panoramic images, the probability of contact between the IMTM and the IAC increased. CBCT is required in these cases. These findings are in agreement with previous studies. A significant association was found between mandible shape and third molar impaction type in this study, a finding that differs from previous studies. Further studies are necessary to determine the association between mandible shape and type of impaction for mandibular third molars. Conclusion: CBCT is required in the preoperative assessment of IMTM when darkening of the roots and interruption of the white line are observed on panoramic images.

## Abbreviations

DPR: Digital panoramic radiography; CBCT: Cone-beam computed tomography; IAC: Inferior alveolar canal; IAN: Inferior alveolar nerve; IMTM: impacted mandibular third molar; 3D: Three-dimensional.

## Competing interests

The authors declare that they have no competing interests.

## Authors’ contributions

IP formulated the conception and design of the study, participated in data acquisition and interpretation of data analysis and prepared the manuscript. CS contributed to data acquisition and interpretation. MTA supervised interpretation, edited, wrote and gave final approval to the manuscript. ZFZ contributed to data acquisition and interpretation. All authors read and approved the final manuscript.

## Pre-publication history

The pre-publication history for this paper can be accessed here:

http://www.biomedcentral.com/1472-6831/14/71/prepub

## References

[B1] Libersa P Savignat M Tonnel A Neurosensory disturbances of the inferior alveolar nerve: a retrospective study of complaints in a 10-year period J Oral Maxillofac Surg 2007 65 1486 1489 10.1016/j.joms.2007.03.023 17656272

[B2] Better H Abromowitz I Shlomi B Kahn A Levy Y Shaham A Chashu G The presurgical workup before third molar surgery: how much is enough? Sensory nerve impairment following mandibular third molar surgery J Oral Maxillofac Surg 2004 62 689 692 10.1016/j.joms.2003.12.009 15170279

[B3] Roeder F Wachtlin D Schulze R Necessity of 3D visualization for the removal of lower wisdom teeth: required sample size to prove non-inferiority of panoramic radiography compared to CBCT Clin Oral Investig 2012 16 699 706 10.1007/s00784-011-0553-8 21519882

[B4] Hillerup S Iatrogenic injury to oral branches of the trigeminal nerve: records of 449 cases Clin Oral Investig 2007 11 133 142 10.1007/s00784-006-0089-5 17186310

[B5] Blondeau F Daniel NG Extraction of IMTM: postoperative complications and their risk factors J Can Den Assoc 2007 73 325 17484797

[B6] Rood JP Permanent damage to inferior alveolar and lingual nerves during the removal of IMTM. Comparison of two methods of bone removal Br Dent J 1992 172 108 110 10.1038/sj.bdj.4807777 1739507

[B7] Gülicher D Gerlach KL Sensory impairment of the lingual and inferior alveolar nerves following removal of IMTM Int J Oral Maxillofac Surg 2001 30 306 312 10.1054/ijom.2001.0057 11518353

[B8] Eyrich G Seifert B Matthews F Matthiessen U Heusser CK Kruse AL Obwegeser JA Lübbers HT 3-Dimensional imaging for lower third molars: is there an implication for surgical removal? J Oral Maxillofac Surg 2011 69 1867 1872 10.1016/j.joms.2010.10.039 21419547

[B9] Valmaseda-Castellón E Berini-Aytés L Gay-Escoda C Inferior alveolar nerve damage after lower third molar surgical extraction: a prospective study of 1117 surgical extractions Oral Surg Oral Med Oral Pathol Oral Radiol Endod 2001 92 377 383 10.1067/moe.2001.118284 11598570

[B10] Xu GZ Yang C Fan XD Yu CQ Cai XY Wang Y He D Anatomic relationship between impacted third mandibular molar and the mandibular canal as the risk factor of inferior alveolar nerve injury Br J Oral Maxillofac Surg 2013 51 e215 e219 10.1016/j.bjoms.2013.01.011 23411471

[B11] Kipp DP Goldstein BH Weiss WW Jr Dysesthesia after mandibular third molar surgery: a retrospective study and analysis of 1,377 surgical procedures JADA 1980 100 185 192 6928147 10.14219/jada.archive.1980.0074

[B12] Monaco G Montevecchi M Bonetti GA Gatto MR Checchi L Reliability of panoramic radiography in evaluating the topographic relation nship between the mandibular canal and impacted third molars JADA 2004 135 312 318 15058618 10.14219/jada.archive.2004.0179

[B13] Nakayama K Nonoyama M Takaki Y Kagawa T Yuasa K Izumi K Ozeki S Ikebe T Assessment of the relationship between IMTM and inferior alveolar nerve with dental 3-dimensional computed tomography J Oral Maxillofac Surg 2009 67 2587 2591 10.1016/j.joms.2009.07.017 19925976

[B14] Sedaghatfar M August MA Dodson TB Panoramic radiographic findings as predictors of inferior alveolar nerve exposure following third molar extraction J Oral Maxillofac Surg 2005 63 3 7 15635549 10.1016/j.joms.2004.05.217

[B15] Lübbers HT Matthews F Damerau G Kruse AL Obwegeser JA Grätz KW Eyrich GK No plane is the best one-the volume is! Oral Surg Oral Med Oral Pathol Oral Radiol 2012 113 421 22676832 10.1016/j.tripleo.2011.05.046

[B16] Flygare L Ohman A Preoperative imaging procedures for lower wisdom teeth removal Clin Oral Investig 2008 12 291 302 10.1007/s00784-008-0200-1 18446390

[B17] Ghaeminia H Meijer GJ Soehardi A Borstlap WA Mulder J Bergé SJ Position of the impacted third molar in relation to the mandibular canal. Diagnostic accuracy of cone beam computed tomography compared with panoramic radiography Int J Oral Maxillofac Surg 2009 38 964 971 10.1016/j.ijom.2009.06.007 19640685

[B18] Feifel H Riediger D Gustorf-Aeckerle R High resolution computed tomography of the inferior alveolar and lingual nerves Neuroradiol 1994 36 236 238 10.1007/BF00588141 8041450

[B19] Lübbers HT Matthews F Damerau G Kruse AL Obwegeser JA Gratz KW Eyrich GK Anatomy of impacted lower third molars evaluated by computerized tomography: is there an indication for 3-dimensional imaging Oral Surg Oral Med Oral Pathol Oral Radiol Endod 2011 111 547 550 10.1016/j.tripleo.2010.06.010 20952229

[B20] Honey OB Scarfe WC Hilgers MJ Klueber K Silveira AM Haskell BS Farman AG Accuracy of cone-beam computed tomography imaging of the temporomandibular joint: comparisons with panoramic radiology and linear tomography Am J Orthod Dentofacial Orthop 2007 132 429 438 10.1016/j.ajodo.2005.10.032 17920495

[B21] Momin MA Matsumoto K Ejima K Asaumi R Kawai T Arai Y Honda K Yosue T Correlation of mandibular impacted tooth and bone morphology determined by cone beam computed topography on a premise of third molar operation Surg Radiol Anat 2013 35 311 318 10.1007/s00276-012-1031-y 23143074

[B22] Suomalainen A Ventä I Mattila M Turtola L Vehmas T Peltola JS Reliability of CBCT and other radiographic methods in preoperative evaluation of lower third molars Oral Surg Oral Med Oral Pathol Oral Radiol Endod 2010 109 276 284 10.1016/j.tripleo.2009.10.021 20123411

[B23] Ghaeminia H Meijer GJ Soehardi A Borstlap WA Mulder J Vlijmen OJ Bergé SJ Maal TJ The use of cone beam CT for the removal of wisdom teeth changes the surgical approach compared with panoramic radiography: a pilot study Int J Oral Maxillofac Surg 2011 40 834 839 10.1016/j.ijom.2011.02.032 21507612

[B24] Neves FS Souza TC Almeida SM Haiter-Neto DQ Freitas FN Bóscolo FN Correlation of panoramic radiography and cone beam CT findings in the assessment of the relationship between IMTM and the mandibular canal Dentomaxillofac Radiol 2012 41 553 557 10.1259/dmfr/22263461 22282507 PMC3608375

[B25] Lübbers HT Kruse AL Obwegeser JA Gratz KW Eyrich G Oblique high resolution tomography: The ideal plane for visualization of the gonial section of the mandibular canal and its related structures? J Healthcare Engineering 2012 3 87 104 10.1260/2040-2295.3.1.87

[B26] Rood JP Shehab BA The radiological prediction of inferior alveolar nerve injury during third molar surgery Br J Oral Maxillofac Surg 1990 28 20 25 10.1016/0266-4356(90)90005-6 2322523

[B27] Selvi F Dodson TB Nattestad A Robertson K Tolstunov L Factors that are associated with injury to the inferior alveolar nerve in high-risk patients after removal of third molars Br J Oral Maxillofac Surg 2013 51 868 873 10.1016/j.bjoms.2013.08.007 24012054

[B28] Guerrero ME Botetano R Beltran J Horner K Jacobs R Can preoperative imaging help to predict postoperative outcome after wisdom tooth removal? A randomized controlled trial using panoramic radiography versus cone-beam CT Clin Oral Investig 2014 18 335 342 10.1007/s00784-013-0971-x 23494455

[B29] Gomes AC Vasconcelos BC Silva ED Caldas Ade F Jr Pita Neto IC Sensitivity and specificity of pantomography to predict inferior alveolar nerve damage during extraction of impacted lower third molars J Oral Maxillofac Surg 2008 66 256 259 10.1016/j.joms.2007.08.020 18201605

[B30] Khan I Halli R Gadre P Gadre KS Correlation of panoramic radiographs and spiral CT scan in the preoperative assessment of intimacy of the inferior alveolar canal to IMTM J Craniofac Surg 2011 22 566 570 10.1097/SCS.0b013e3182077ac4 21403569

[B31] Jerjes W El-Maaytah M Swinson B Upile T Thompson G Gittelmon S Baldwin D Hadi H Vourvachis M Abizadeh N Al Khawalde M Hopper C Inferior alveolar nerve injury and surgical difficulty prediction in third molar surgery: the role of dental panoramic tomography J Clin Dent 2006 17 122 130 17240930

[B32] Tantanapornkul W Okouchi K Fujiwara Y Yamashiro M Maruoka Y Ohbayashi N Kurabayashi TA A comparative study of cone-beam computed tomography and conventional panoramic radiography in assessingthe topographic relationship between the mandibular canal and impacted third molars Oral Surg Oral Med Oral Pathol Oral Radiol Endod 2007 103 253 259 10.1016/j.tripleo.2006.06.060 17234544

[B33] Nakagawa Y Ishii H Nomura Y Watanabe NY Hoshiba D Kobayashi K Ishibashi K Third molar position: reliability of panoramic radiography J Craniofac Surg 2007 65 1303 1308 10.1016/j.joms.2006.10.028 17577493

[B34] Jung YH Nah KS Cho BH Correlation of panoramic radiographs and cone beam computed tomography in the assessment of a superimposed relationship between the mandibular canal and impacted third molars Imaging Sci Dent 2012 42 121 127 10.5624/isd.2012.42.3.121 23071961 PMC3465753

[B35] Jhamb A Dolas RS Pandilwar PK Mohanty S Comparative efficacy of spiral computed tomography and orthopanthomography in preoperative detection of relation of inferior alveolar neurovascular bundle to the impacted mandibular third molar J Oral Maxillofac Surg 2009 67 58 66 19070749 10.1016/j.joms.2008.06.014

[B36] Szalma J Lempel E Jeges S Olasz L Darkening of third molar roots: panoramic radiographic associations with inferior alveolar nerve exposure J Oral Maxillofac Surg 2011 69 1544 1549 10.1016/j.joms.2010.09.009 21292368

[B37] Msagati F Simon EN Owibingire S Pattern of occurrence and treatment of impacted teeth at the Muhimbili National Hospital, Dar es Salaam, Tanzania BMC Oral Health 2013 13 37 10.1186/1472-6831-13-37 23914842 PMC3750328

[B38] Syed KB Zaheer KB Ibrahim M Bagi MA Assiri MA Prevalence of impacted molar teeth among Saudi population in asir region, Saudi Arabia - a retrospective study of 3 years J Int Oral Health 2013 5 43 47 10.1093/inthealth/ihs037 24155577 PMC3768082

[B39] Watanabe H Mohammad Abdul M Kurabayashi T Aoki H Mandible size and morphology determined with CT on a premise of dental implant operation Surg Radiol Anat 2010 32 343 349 10.1007/s00276-009-0570-3 19812884

[B40] Quirynen M Mraiwa N van Steenberghe D Jacobs R Morphology and dimension of the mandibular jaw bone in the interforaminal region in patients requiring implants in the distal areas Clin Oral Imp Res 2003 14 280 285 10.1034/j.1600-0501.2003.140305.x 12755778

[B41] Lin MH Mau LP Cochran DL Shieh YS Huang PH Huang RY Risk assessment of inferior alveolar nerve injury for immediate implant placement in the posterior mandible: A virtual implant placement study J Dent 2014 5712 349 347 10.1016/j.jdent.2013.12.014 24394585

